# Riociguat prevents hyperoxia-induced lung injury and pulmonary hypertension in neonatal rats without effects on long bone growth

**DOI:** 10.1371/journal.pone.0199927

**Published:** 2018-07-10

**Authors:** Keyur Donda, Ronald Zambrano, Younghye Moon, Justin Percival, Ruben Vaidya, Fredrick Dapaah-Siakwan, Shihua Luo, Matthew R. Duncan, Yong Bao, Luqing Wang, Ling Qin, Merline Benny, Karen Young, Shu Wu

**Affiliations:** 1 Pediatrics and Batchelor Children’s Research Institute, University of Miami School of Medicine, Miami, Florida, United States of America; 2 Molecular and Cellular Pharmacology, University of Miami Miller School of Medicine, Miami, Florida, United States of America; 3 Department of Orthopedic Surgery, School of Medicine, University of Pennsylvania, Philadelphia, Pennsylvania, United States of America; Center of Pediatrics, GERMANY

## Abstract

Bronchopulmonary dysplasia (BPD) remains the most common and serious chronic lung disease of premature infants. Severe BPD complicated with pulmonary hypertension (PH) increases the mortality of these infants. Riociguat is an allosteric soluble guanylate cyclase stimulator and is approved by the FDA for treating PH in adults. However, it has not been approved for use in neonates due to concern for adverse effects on long bone growth. To address this concern we investigated if administration of riociguat is beneficial in preventing hyperoxia-induced lung injury and PH without side effects on long bone growth in newborn rats. Newborn rats were randomized to normoxia (21% O_2_) or hyperoxia (85% O_2_) exposure groups within 24 hours of birth, and received riociguat or placebo by once daily intraperitoneal injections during continuous normoxia or hyperoxia exposure for 9 days. In the hyperoxia control group, radial alveolar count, mean linear intercept and vascular density were significantly decreased, the pathological hallmarks of BPD, and these were accompanied by an increased inflammatory response. There was also significantly elevated vascular muscularization of peripheral pulmonary vessels, right ventricular systolic pressure and right ventricular hypertrophy indicating PH. However, administration of riociguat significantly decreased lung inflammation, improved alveolar and vascular development, and decreased PH during hyperoxia by inducing cGMP production. Additionally, riociguat did not affect long bone growth or structure. These data indicate that riociguat is beneficial in preventing hyperoxia-induced lung injury and PH without affecting long bone growth and structure and hence, suggests riociguat may be a potential novel agent for preventing BPD and PH in neonates.

## Introduction

Bronchopulmonary dysplasia (BPD) is the most common and serious chronic lung disease of premature infants [1]. Over the past four decades, the incidence of this disease has significantly increased as a result of the improved survival of very low birth weight infants. BPD develops in about 40% of preterm infants with birth weight <1000 g, accounting for approximately 15,000 new cases annually in the US [2, 3]. The cost for treating BPD in the US is approximately $3 billion/year. The lung pathology of BPD is characterized by decreased alveolarization and vascular growth [1]. The decreased vascular formation and increased vascular remodeling can lead to the development of pulmonary hypertension (PH) [4–6]. The mortality rate of severe BPD complicated with PH is as high as 50% [7]. Unfortunately, there is no effective therapy for BPD with PH due to its multifactorial etiology and poorly understood disease processes that impact not only alveolar structure but also the vasculature.

The nitric oxide (NO)-soluble guanylate cyclase (sGC)-cyclic guanosine monophosphate (cGMP) pathway plays an important role in regulating vasodilation. It also has anti-proliferative and anti-inflammatory properties [8]. Two agents that target this pathway, inhaled NO (iNO) and sildenafil, are currently used in patients with BPD and PH [9–12]. INO, a potent pulmonary vasodilator, stimulates sGC and increases levels of cGMP. It is widely used for acute PH because of its short half-life. But there are two major limitations. First, it’s very expensive and second, it’s not practical for long-term treatment on an outpatient basis. Sildenafil is a selective inhibitor of phosphodiesterase type 5 (PDE-5) which increases levels of cGMP by preventing its degradation. Sildenafil is used off-label for more long-term management of PH in BPD since it is available in both IV and oral forms [7]. However, at the current time there is no formal evidence from randomized controlled trials supporting the benefit of sildenafil in BPD with PH. Moreover a recent study reported a dose-related increase in mortality with sildenafil in children with PH secondary to idiopathic PH and congenital heart disease [13]. Thus the Food and Drug Administration (FDA) has issued an warning for its judicious use in children with PH [13]. Other potential vasodilators such as bosentan, an antagonist of the endothelin receptor, and iloprost, a prostacyclin analogue that stimulates adenylate cyclase and increases cAMP have been used to treat BPD with PH in small case reports [7]. Because of limited options available, severe BPD with PH still remains a common and fatal disease in premature babies. Therefore, there is an urgent need for novel therapeutic agents that can prevent or treat severe BPD with PH.

Recently, the FDA has approved riociguat, a potent stimulator of sGC for treatment of adult pulmonary arterial hypertension [14]. Rather than preventing cGMP degradation like sidenafil, riociguat stimulates sGC activity and increases cGMP production in the presence of low levels of NO or in a NO-independent manner. Its therapeutic potential is superior to iNO, as its use is not complicated by uncontrolled NO-release, development of tolerance after prolonged usage, or non-specific interactions of NO with other biological molecules [8]. Exposure of neonatal lung to oxidative stress reduces NO-sGC-cGMP pathway by oxidizing heme-bound sGC, leading to increased sGC inactivation or degradation [15, 16]. Thus, by stimulating sGC, riociguat could be very beneficial in the setting of BPD with PH in that oxidative stress is a key inducer. However, riociguat is not approved by the FDA for use in neonates or pediatric patients, given the concern that sGC agonists may cause abnormal bone growth [17].

Hyperoxia-induced lung injury in neonatal rodents is widely used as an experimental model for BPD [18]. We have previously shown that chronic hyperoxia exposure induces BPD and PH like changes characterized by alveolar simplification, decreased pulmonary vascular development, excessive pulmonary vascular remodeling, and increased right ventricular hypertrophy (RVH) [19–21]. In this study, we utilized a hyperoxia-induced BPD and PH model in neonatal rats to test the efficacy of riociguat in preventing lung injury and PH. In addition, we also assessed the effects of riociguat on long bone growth and development in these animals.

### Materials

Pregnant Sprague-Dawley rats were purchased from Jackson Laboratory (Bar Harbor, ME). Riociguat was obtained from Medchemexpress (Monmouth Junction, NJ). The following antibodies were used for immunostaining, double immunofluorescence staining and Western blot analyses: a rabbit anti-vonWillebrand factor (vWF) antibody from Dako (Carpinteria, CA); a mouse anti- α-smooth muscle actin (α-SMA) from Sigma (Saint Louis, MI); a rat anti-Mac3 antibody from BD Biosciences (San Jose, CA); rabbit anti-Ki67, anti-inducible nitric oxide synthase (iNOS), anti-chitinase 3-like 3 (Ym1), and anti-NLR family pyrin domain containing 1 (NLRP-1) antibodies from Abcam (Cambridge, MA); rabbit anti-caspase-1 and anti-connective tissue growth factor (CTGF) antibodies, and a goat anti-resistin-like molecule alpha (RELM-α) antibody from Santa Cruz (Dallas, TX)); a goat anti-IL-1β antibody from R&D System (Minneapolis, MN).

### Animal models and experimental protocol

Pregnant Sprague-Dawley rats were cared for according to NIH guidelines for the use and care of laboratory animals, and the study protocol was approved by the University of Miami Animal Care and Use Committee (protocol number 16–030). Within 24 h after birth, rat pups were randomized into 4 groups: normoxia (RA, 21% O_2_) plus placebo (PL), normoxia plus riociguat (Rio), hyperoxia (85% O_2_) plus placebo, and hyperoxia plus riociguat. BPD was induced by keeping newborn rats in a chamber with continuous exposure to 85% O_2_ and the oxygen level inside the chamber was monitored continuously with a Ceramatec (MAXO2) oxygen analyzer. Nursing dams were rotated between normoxia and hyperoxia groups once every 48 h to prevent oxygen toxicity in the dams. Rat pups in normoxia and hyperoxia groups received riociguat or placebo (equal volume) by daily intraperitoneal (IP) injection from postnatal day 1 (P1) to P9. In the riociguat groups, animal received riociguat 10 mg/kg on P1, 5 mg/kg on P2, and then 2.5 mg/kg daily from P3 to P9. Riociguat was dissolved in a vehicle consisting of a mixture of dimethyl sulfoxide (Sigma-Aldrich, St. Louis, MO, US), Transcutol (Sigma-Aldrich, St. Louis, MO, US), and PEG400 (Merck, Darmstadt, Germany) in a ratio of 1:49.5:49.5%. The same vehicle was used as a placebo solution. On P10, pups were anesthetized by 0.1% isoflurane, tracheotomized and cannulized, and then sacrificed for analyses.

### Lung histology and morphometry

Lungs were infused with 4% paraformaldehyde via a tracheal catheter at 20 cm H_2_O pressure for 5 min, fixed overnight, embedded in paraffin wax and then sectioned. Hematoxylin and eosin (H&E) staining was performed for lung histology, radial alveolar count (RAC) and mean linear intercept (MLI) measurements as previously described [22, 23].

### Assessment of lung inflammation

Macrophage infiltration into alveolar airspaces was assessed by performing immunostaining with a Mac3 antibody (total macrophage marker) on lung tissue sections. The numbers of Mac3 positive cells in the alveolar airspaces were counted as described previously [23]. Macrophage phenotype was assessed by immunostaining for iNOS, a M1 marker, and Ym1 and RELM-α, M2 markers [24–26].

### Pulmonary vascular morphometry

Pulmonary vascular density was determined by the average number of vWF stained vessels (< 50 μm in diameter) from 10 random images on each lung section [18, 22, 23].

### Assessment of pulmonary vascular remodeling

Double immunofluorescence staining was performed as previously described using anti-vWF staining as an endothelial cell specific marker and antiα-SMA staining as a marker for vascular smooth muscle cells [18, 22, 23]. Twenty peripheral pulmonary vessels (<50 μm in diameter) were assessed for their degree of excessive muscularization (>50% of vessel circumference α-SMA positive) and medial wall thickness (MWT) as previously described [18, 22, 23]. Double immunofluorescence staining for Ki67, a nuclear proliferation marker and α-SMA was performed to assess vascular smooth muscle proliferation in vessels that are <50 μm in diameter [22, 23].

### Assessment of pulmonary hypertension

Right ventricular systolic pressure (RVSP) and right ventricle (RV) to left ventricle (LV) plus septum weight ratios (RV/LV+S) were determined as indices for PH [18, 22, 23]. For RVSP measurement a 25-gauge needle fitted to a pressure transducer was inserted into the RV. Pressure levels were recorded on a Gould polygraph. Afterwards right ventricle was dissected from the LV+S for RV/LV+S weight ratio assessment as a marker for right ventricular hypertrophy (RVH) [18, 22, 23].

### Western blot analysis

Total protein was extracted from frozen lung tissues with a RIPA buffer according to manufacturer’s instructions (Santa Cruz, Dallas, TX). Western blot analysis was performed to assess target protein expression in lung homogenates as previously described [22, 23].

### Assessment of cGMP level

Steady state cGMP concentrations were measured in lung homogenates by ELISA, catalog number K020-H1 from Arbor Assays (Ann Arbor, MI), according to the manufacturer’s instructions as previously described [27].

### Assessment of bone growth

Pups were euthanized on P10 and their tibiae (n = 5/group) were harvested for μCT analyses (microCT 35, Scanco Medical AG, Brüttisellen, Switzerland). Briefly, a scout view of the entire tibia was performed to measure the length from the upper extremity to the tibiofibular junction at ankle site. The tibial proximal end was scanned at 6 μm isotropic voxel size. All images were first smoothed by a Gaussian filter (sigma = 1.2, support = 2.0) and then threshold corresponding to 22% of the maximum available range of image gray scale values. The images of metaphyseal region (0–0.9 mm below the lowest point of growth plate) were contoured for trabecular bone analysis. Geometric trabecular volumetric bone mineral density (vBMD), bone volume fraction (BV/TV), trabecular thickness (Tb.Th), trabecular separation (Tb.Sp), trabecular number (Tb.N), and structure model index (SMI), were calculated by 3D standard microstructural analysis [28].

### Statistical analysis

Data were expressed as means ± SD and statistical comparisons were performed by analysis of variance (ANOVA) followed by Holm-Sidac post *hoc* analysis. A *P-*value < 0.05 was considered significant.

## Results

### Riociguat prevents alveolar disruption in hyperoxia exposed animals

**Fig 1:**
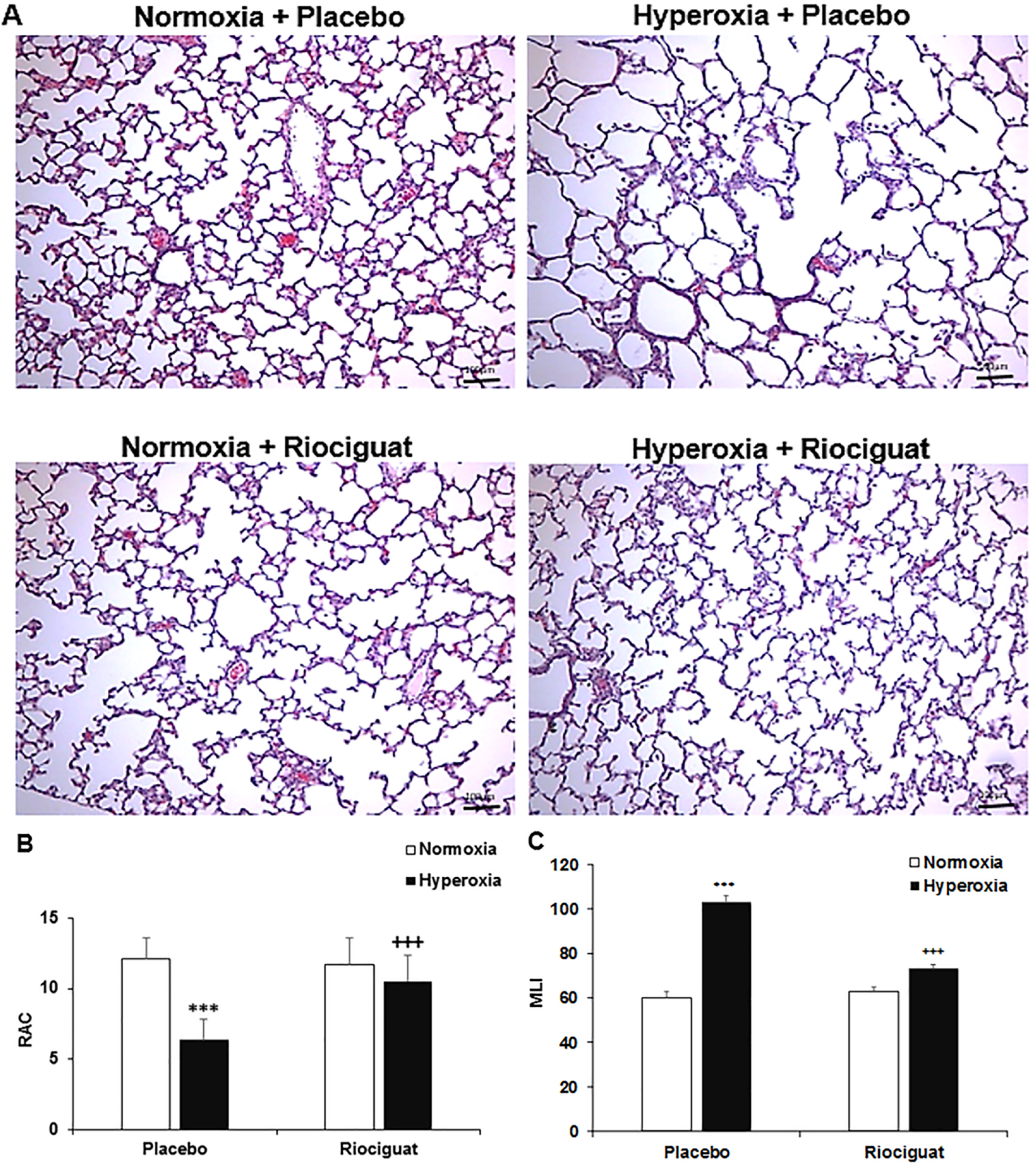
Riociguat prevents hyperoxia-impaired alveolarization. (**A**) H & E stained lung histology. Hyperoxia exposure in the presence of placebo decreased radial alveolar count (RAC) (**B**) and increased mean linear intercept (MLI) (**C**) as compared with normoxia. Administration of riociguat increased RAC and decreased MLI during hyperoxia. ***P < 0.001 compared with normoxia; ^+++^*P* < 0.001 compared with hyperoxia + placebo (n = 6/group). Scale bar: 100 μm.

On histological examination, lungs of animals exposed to hyperoxia and placebo showed simplified alveoli characterized by larger, fewer and less complex alveoli as compared to animals in the normoxia plus placebo group. The lungs from hyperoxia plus riociguat group appeared to have smaller and more complex alveoli as compared to the placebo treated hyperoxia group (**Fig 1A**). Morphometric analyses including RAC and MLI were performed and showed that the hyperoxia plus placebo lungs have decreased RAC (**Fig 1B**) and increased MLI (**Fig 1C**) as compared to the normoxia groups, suggesting poor alveolarization. However, pups who received riociguat during hyperoxia exposure, showed increased RAC and decreased MLI as compared to the hyperoxia exposed placebo treated group (10.52 ± 0.611 vs. 6.37 ± 0.826, *P* < 0.001) (**Fig 1B and 1C**), indicating better alveolar development.

### Riociguat prevents ablated pulmonary vascular development in hyperoxia exposed animals

**Fig 2:**
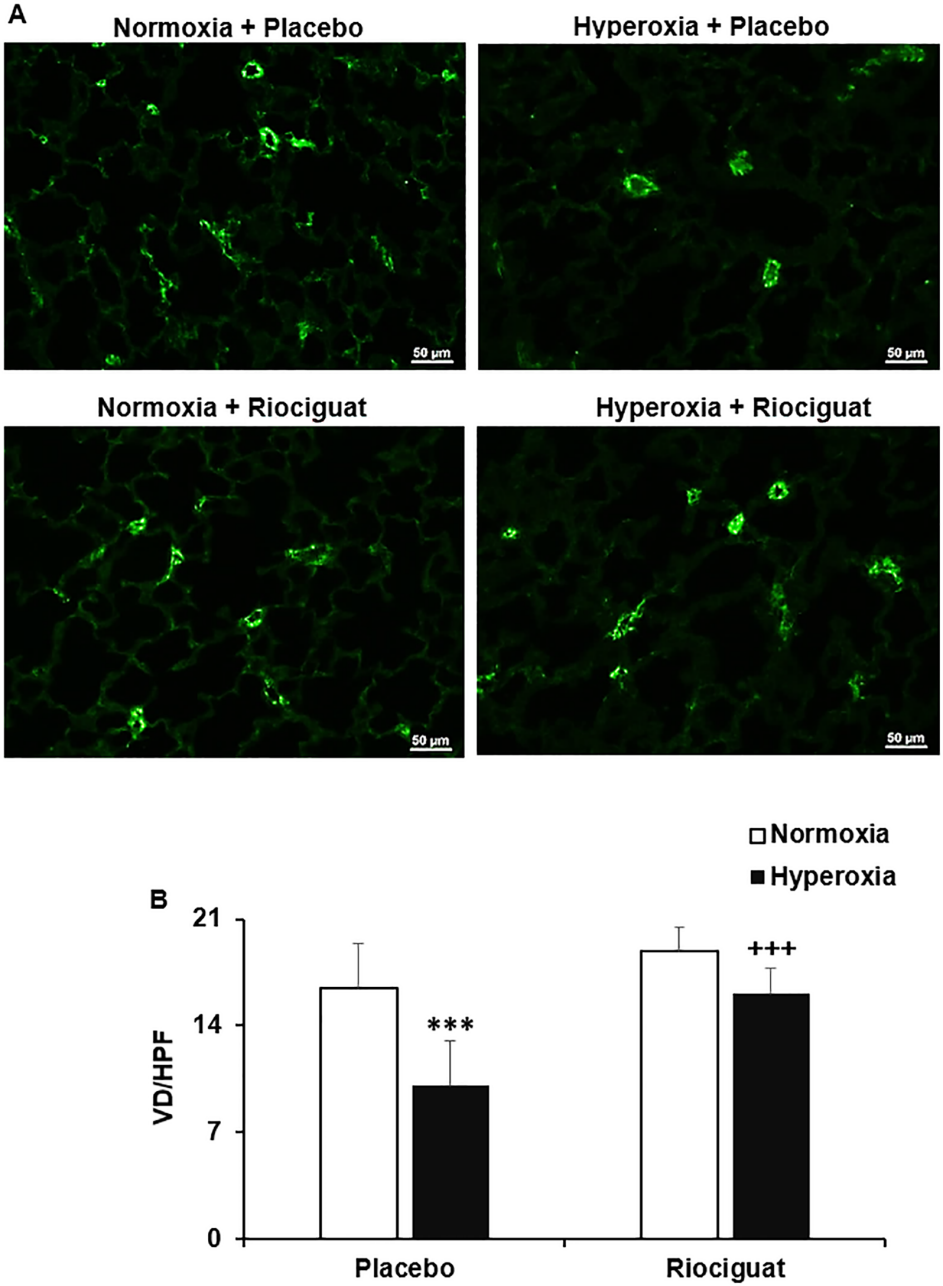
Riociguat prevents hyperoxia-ablated vascular development. (**A**) Immunofluorescence staining for von-Willebrand factor (vWF) (green signal). Vascular density (VD) was determined by counting vWF-positive vessels (<50 μm in diameter) on 10 random high-power field (HPF) images from each lung section. (**B**) Hyperoxia exposure in the presence of placebo significantly decreased VD as compared with normoxia group. Administration of riociguat increased VD in hyperoxia exposed lungs. ***P < 0.001 compared with normoxia; ^+++^*P* < 0.001 compared with hyperoxia + placebo (n = 6/group). Scale bar: 50 μm.

In comparison with normoxia + placebo controls, vascular density was significantly decreased in the placebo treated hyperoxia group (16.433 ± 2.97 vs. 10.067 ± 1.573, *P* < 0.001). Conversely, riociguat administration during hyperoxia exposure significantly increased vascular density in comparison with placebo treated hyperoxia animals (16.1 ± 1.307 vs. 10.067 ± 1.573, *P* < 0.001) (**Fig 2**).

### Riociguat decreases pulmonary vascular remodeling in hyperoxia exposed animals

**Fig 3:**
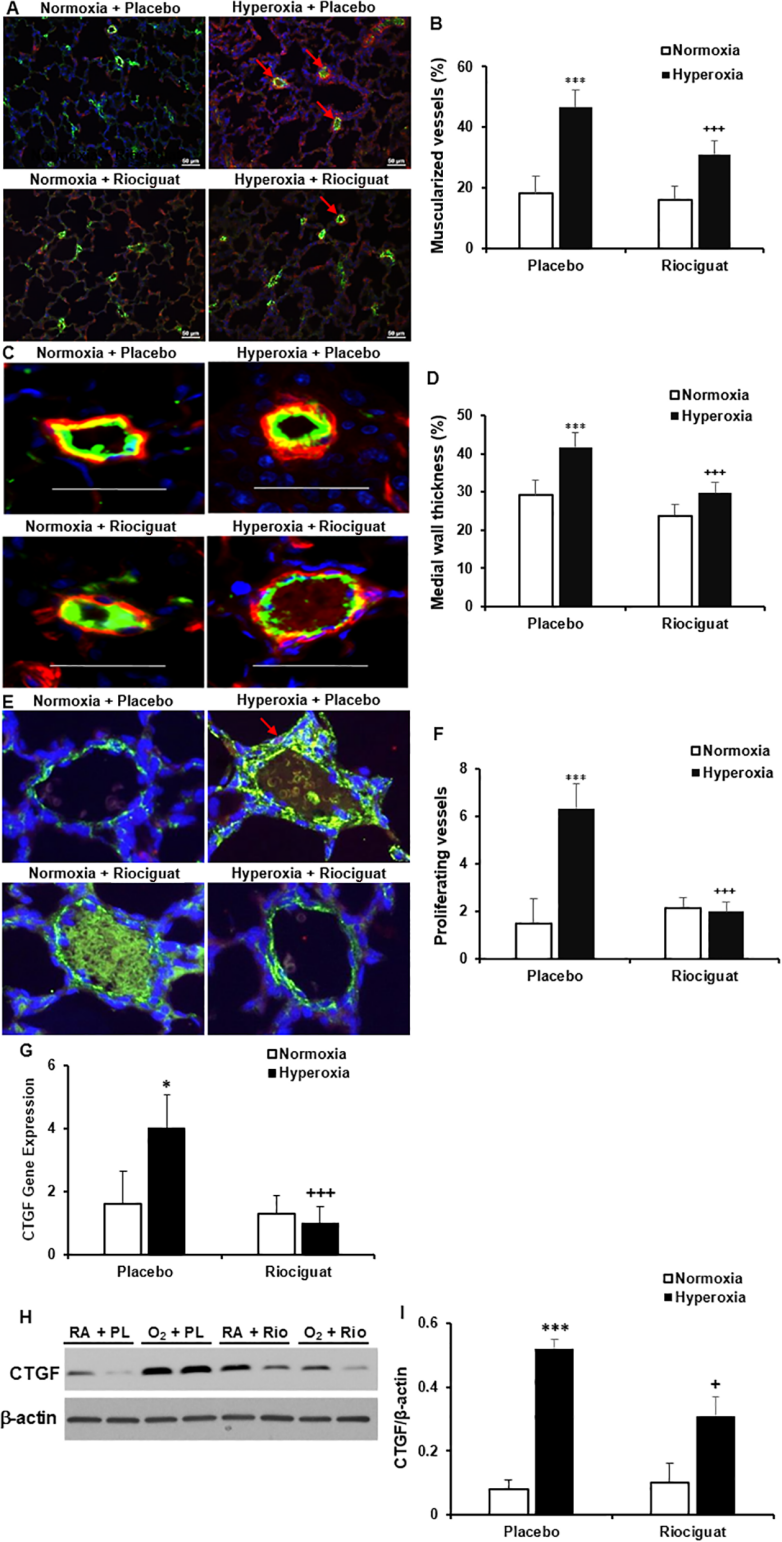
Riociguat decreases hyperoxia-induced vascular remodeling. (**A**, **C**) Double immunofluorescence staining for vWF (green signal) and α-SMA (red signal) plus DAPI nuclear stain (blue signal). (**B**) Hyperoxia exposure in the presence of placebo increased muscularization of peripheral pulmonary vessels (<50 μm in diameter) as compared with normoxia group (red arrow). Administration of riociguat decreased muscularized vessels in hyperoxia exposed lungs. (**D**) Hyperoxia increased medial wall thickness (MWT) in presence of placebo as compared with normoxia group. Riociguat administration significantly decreased MWT in hyperoxia group. ***P < 0.001 compared with normoxia; ^+++^*P* < 0.001 compared with hyperoxia + placebo (n = 6/group). Scale bar: 50 μm. (**E**) Double immunofluorescence staining with Ki67 (red arrow) and α-SMA (green signal) plus DAPI nuclear staining (blue signal). (**F**) Hyperoxia exposure in the presence of placebo increased vascular proliferation as compared with normoxia group. Administration of riociguat decreased vascular proliferation. ***P < 0.001 compared with normoxia; ^+++^*P* < 0.001 compared with hyperoxia + placebo (n = 6/group). (**G**) CTGF gene expression was up-regulated by hyperoxia and it was down-regulated by riociguat. *P < 0.05 compared with normoxia; ^+++^*P* < 0.001 compared with hyperoxia + placebo (n = 6/group). (**H**) Representative Western blots of CTGF and β-actin. (**I**). Expression of CTGF was increased by hyperoxia, while administration of riociguat decreased CTGF expression in hyperoxia exposed lungs. ***P < 0.001 compared with normoxia; ^+^*P* < 0.05 compared with hyperoxia + placebo (n = 6/group). RA: room air, normaxia; O_2_: hyperoxia; PL: placebo; Rio: riociguat.

Hyperoxia exposure in placebo treated rats significantly increased the percentage of muscularized peripheral pulmonary arterioles (the vessels with >50% muscularization), MWT, and vascular smooth muscle proliferation in comparison with normoxia + placebo controls (46.5 ± 8.84% vs. 18.1 ± 5.73%, *P* < 0.001; 0.416 ± 0.024 vs. 0.291 ± 0.038 μm, *P* < 0.001; 6.333 ± 1.003 vs. 1.5 ± 1.049, *P* < 0.001, respectively). However, riociguat administration during hyperoxia exposure significantly decreased vascular remodeling as was evident by decreased numbers of muscularized pulmonary arterioles, MWT, and vascular smooth muscle proliferation (<50 μm in diameter) (31 ± 6.73 vs. 46.5. ± 8.84%, *P* < 0.001; 0.297 ± 0.023 vs. 0.416 ± 0.024 μm, *P* < 0.001; 2 ± 1.265 vs. 6.333 ± 1.003, *P* < 0.001, respectively) (**Fig 3A–3F**). CTGF is a known fibrotic cytokine that induces pulmonary vascular remodeling and PH (18, 22). Treatment with riociguat decreased CTGF gene and protein expression in hyperoxia-exposed lungs (**Fig 3G–3I**).

### Riociguat decreases hyperoxia-induced pulmonary hypertension

**Fig 4:**
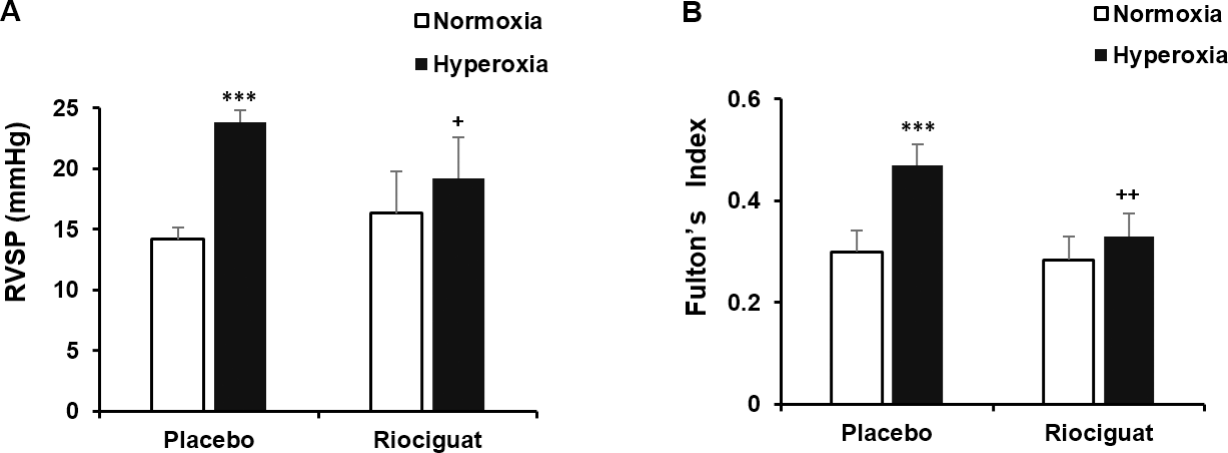
Riociguat reduces hyperoxia-induced PH. (**A**) Right ventricular systolic pressure (RVSP) was significantly increased in the hyperoxia + placebo treated group as compared with normoxia group. Riociguat administration significantly decreased RVSP during hyperoxia. ***P < 0.001 compared with normoxia; ^+^*P* < 0.05 compared with hyperoxia + placebo (n = 6/group). (**B**) Right ventricular hypertrophy (RVH), also known as the Fulton’s index, was determined by the weight ratio of right ventricle (RV) to left ventricle + septum (LV + S). Hyperoxia exposed animals in the presence of placebo showed significant RVH as compared with normoxia group. Administration of riociguat decreased RVH in hyperoxia exposed lungs. ***P < 0.001 compared with normoxia; ^++^*P* < 0.01 compared with hyperoxia + placebo (n = 6/group).

RV/LV+S (also known as Fulton’s index), a marker for RVH, and RVSP were assessed as surrogate markers of PH to determine the effect of riociguat on PH. In comparison to placebo treated normoxic animals, hyperoxia induced significant PH in placebo treated animals as was evident by the significant elevation in RVSP and increased RV/LV+S (23.833 ± 4.119 mmHg vs. 14.167 ± 0.983 mmHg, *P* < 0.001 and 0.469 ± 0.122 vs. 0.299 ± 0.043, *P* < 0.001, respectively). Daily administration of riociguat to hyperoxia exposed animals significantly decreased their RVSP and RV/LV+S compared to placebo treated hyperoxia animals (19.167 ± 2.229 mmHg vs. 23.833 ± 4.119 mmHg, *P* < 0.05; 0.33 ± 0.066 vs. 0.469 ± 0.122, *P* < 0.01, respectively) (**Fig 4**).

### Effects of riociguat on lung inflammatory response

**Fig 5:**
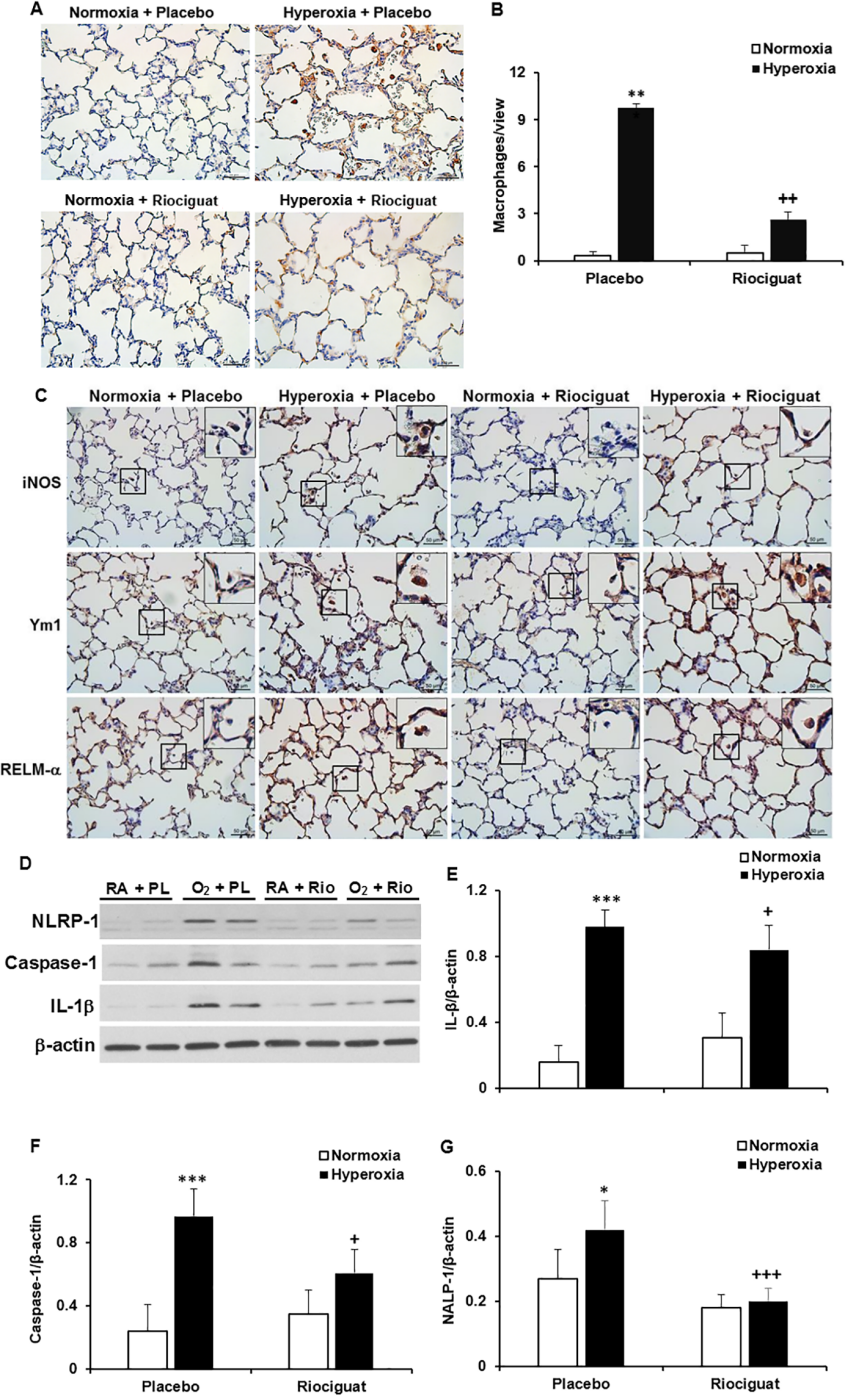
Riociguat reduces and alters hyperoxia-induced lung inflammation. (**A**) Immunostaining for Mac-3, a macrophage marker. (**B**) The alveolar airspace macrophage population was increased by hyperoxia exposure as compared to normoxia. Administration of riociguat decreased macrophage count during hyperoxia. ***P < 0.001 compared with normoxia; ^++^*P* < 0.01 compared with hyperoxia + placebo (n = 6/group). (**C**) Immunostaining for the M1 marker, inducible nitric oxide synthase (iNOS), and M2 markers, chitinase 3-like 3 (Ym1) and resistin-like molecule alpha (RELM-α) showed that in hyperoxia plus placebo lungs, both M1 and M2 polarized macrophages were detected. But, treatment with riociguat decreased only M1 macrophages in hyperoxia-exposed lungs. (**D**) Representative Western blots for NLRP-1, active caspase-1 and active IL-1β. Administration of riociguat decreased hyperoxia-induced lung expression of (**E**) NLRP-1 (***P < 0.001 compared with normoxia; ^+^*P* < 0.05 compared with hyperoxia + placebo), (**F**) active caspase-1 (***P < 0.001 compared with normoxia; ^++^*P* < 0.05 compared with hyperoxia + placebo), and (**G**) active IL-1β (*P < 0.05 compared with normoxia; ^+++^*P* < 0.001 compared with hyperoxia + placebo). RA: room air, normoxia; O_2_: hyperoxia; PL: placebo; Rio: riociguat.

Total macrophage counts, assessed by Mac3 staining were significantly elevated in placebo treated and hyperoxia exposed rats as compared to the normoxia plus placebo group (9.77 ± 7.34 vs. 3.33 ± 2.42, *P* < 0.001). However, riociguat administration reduced total macrophage counts during hyperoxia exposure (2.60 ± 1.83 vs. 9.77 ± 7.34, *P* < 0.01) (**Fig 5A and 5B)**. We assessed the phenotypes of macrophages by immunostaining for the M1 marker, iNOS, and the M2 markers Ym1 and RELM-α. As demonstrated in **Fig 5C**, the macrophages in the two normoxic groups were negative for iNOS, Ym1 and RELM-α, suggesting they are un-polarized macrophages. The macrophages in the hyperoxia plus placebo group were positive for iNOS, Ym1 and RELM-α, indicating both M1 and M2 macrophages are induced by hyperoxia exposure. However, the macrophages in hyperoxia and riociguat exposed lungs were negative for iNOS, but positive for Ym1 and RELM-α, highlighting that riociguat prevents only hyperoxia-induced M1 polarization. Given the importance of NLRP inflammasome and mature IL-1β in clinical and experimental BPD, we further analyzed their expression. Hyperoxia increased expression of NLRP-1 inflammasome components, including NLRP-1 and active caspase-1, and their down-stream effector, mature IL-1β, but treatment with riociguat down-regulated these protein’s expression in the hyperoxic lungs (**Fig 5D–5G**).

### Riociguat iIncreases lung tissue cGMP levels

**Fig 6:**
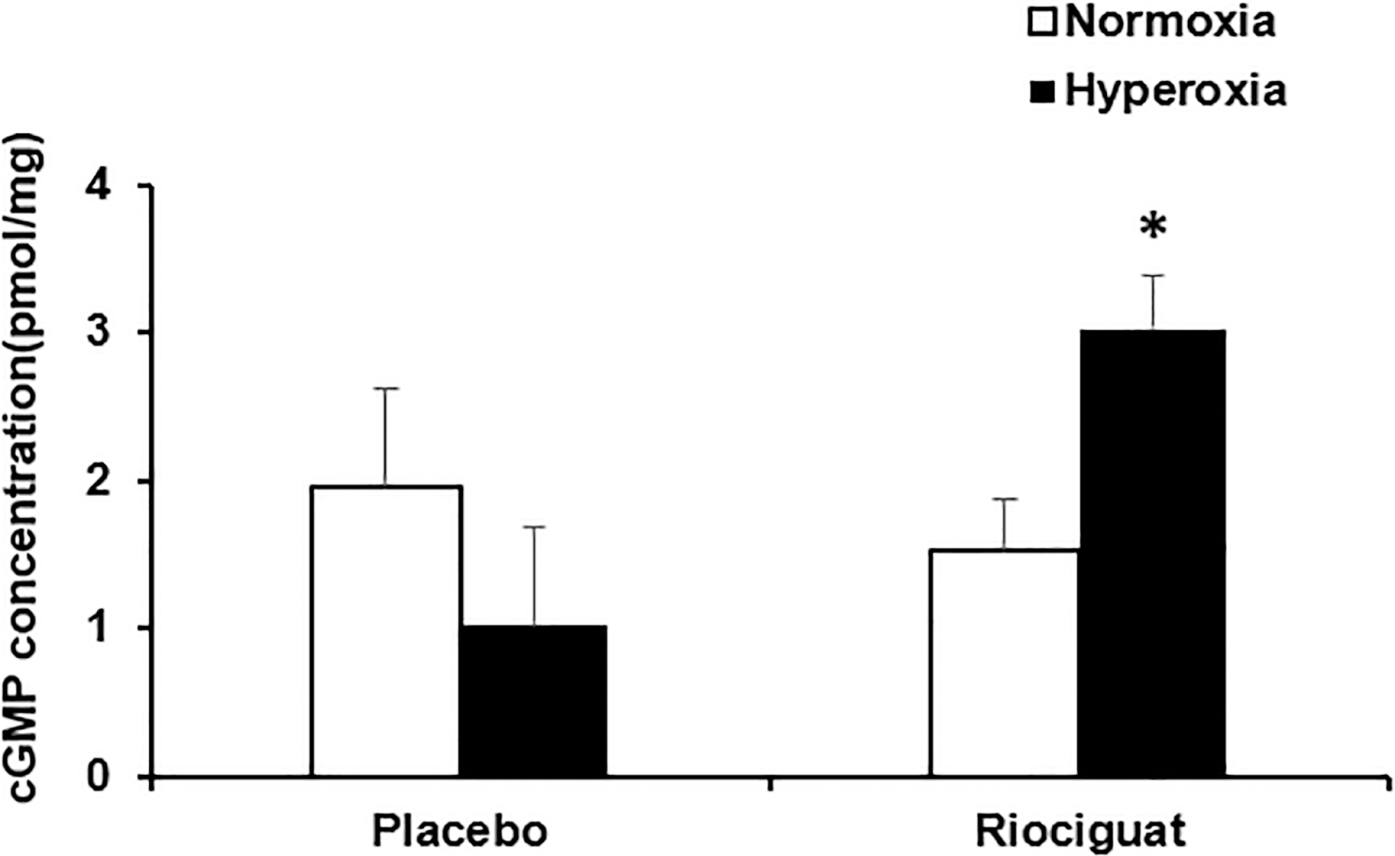
Riociguat increases lung tissue cGMP levels. Riociguat significantly increased cGMP concentration in hyperoxia-exposed rats as compared with placebo treated hyperoxic rats. *P < 0.05 compared with hyperoxia + placebo (n = 4/group).

In the normoxia group, administration of riociguat did not change steady state lung tissue cGMP levels (1.954 ± 1.475 pmol/mg protein vs. 1.517 ± 0.654 pmol/mg protein, P = 0.789). However, in hyperoxia exposed animals, riociguat markedly increased steady state cGMP concentrations in lung tissues as compared to placebo treated animals (3.028 ± 1.703 pmol/mg protein vs. 1.019 ± 0.654 pmol/mg protein, P < 0.05) (**Fig 6**).

### Riociguat doesn’t affect long bone growth and formation

**Fig 7:**
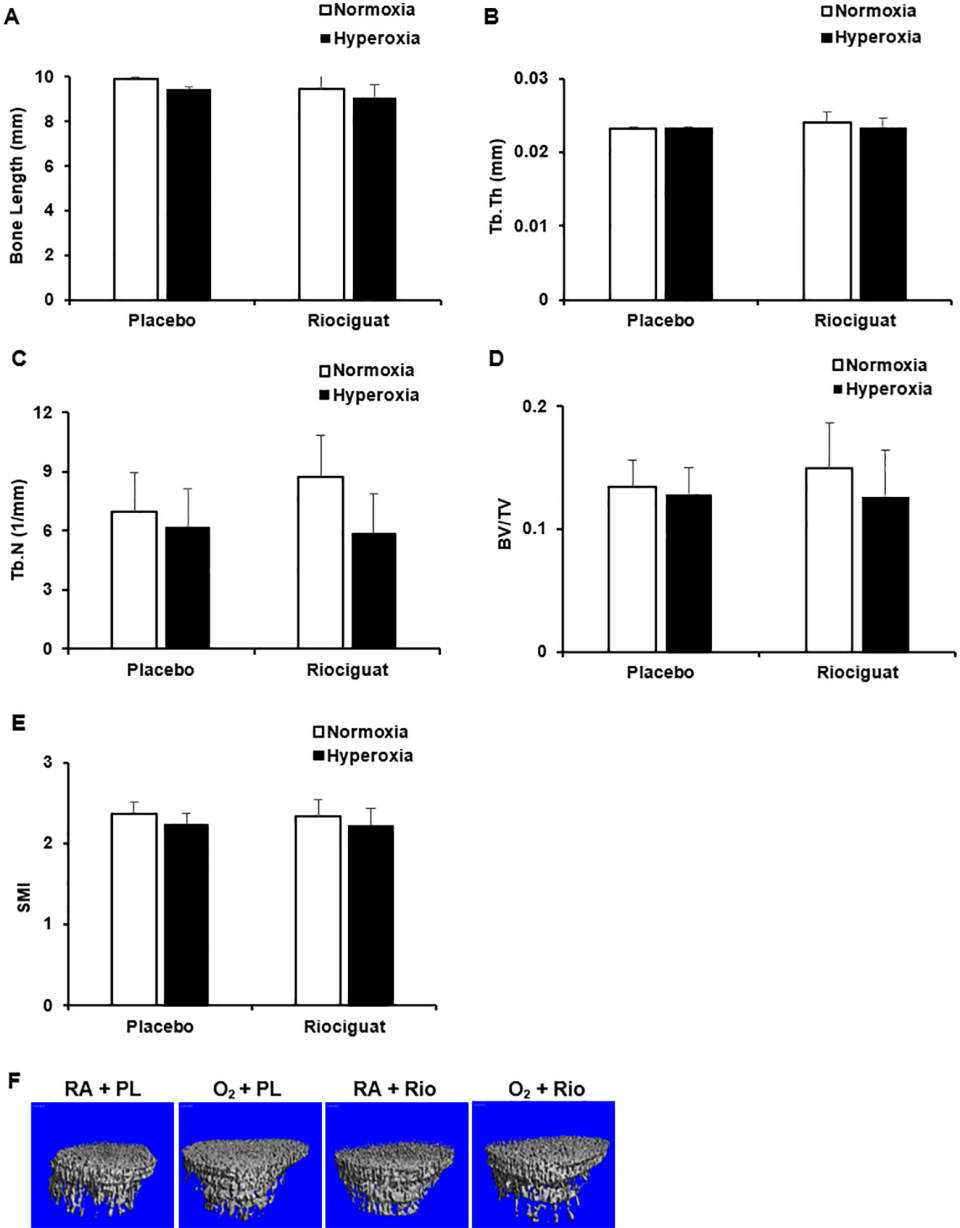
Riociguat does not affect femur growth and structure. (**A**) Bone length. (**B**) Trabecular thickness (Tb.Th). (**C**) Trabecular number (Tb.N). (**D**) Bone volume fraction (BV/TV). (**E**) Structural model index (SMI). (**F**) Representative micro-CT images. n = 5/group.

We found no significant difference in tibial length among four groups with or without hyperoxia and riociguat treatment (**Fig 7A**). In addition, high resolution microCT scans revealed that major structure parameters of metaphyseal trabecular bone, including bone volume fraction (BV/TV), trabecular thickness (Tb.Th), trabecular number (Tb.N), trabecular separation (Tb.Sp), and structure model index (SMI), are also not altered among these groups (**Fig 7B**), suggesting that at this dose and time interval, riociguat doesn’t affect longitudinal growth and structure of long bones.

## Discussion

In this study we demonstrate that riociguat, a sGC stimulator, improves distal lung development and vascular growth, while attenuating pulmonary vascular remodeling leading to prevention of PH in a hyperoxia-induced neonatal rat model of BPD, by increasing production of cGMP in the lung. These beneficial effects of riociquat are correlated with a decreased inflammatory response in hyperoxia-exposed animals. In addition, we did not observe any short-term effects of riociguat on long bone growth and formation. To our knowledge, this is the first study describing the effects of riociguat on lung and vascular development, as well as on PH, and on long bone formation in the neonatal rat model of BPD and PH and therefore, it identifies riociguat as a potential novel therapeutic agent for infants with PH associated with BPD.

Cyclic GMP is a pivotal secondary messenger, regulating vascular contractility, inflammation, smooth muscle cell proliferation, fibrosis and the structural development of the lung [29, 30]. Currently, the two most commonly used agents for PH associated with BPD, iNO and sildenafil, work via increasing intracellular cGMP levels. Inhaled NO is the most popular agent used to acutely relieve PH in mechanically ventilated infants. It exerts its effects by increasing intracellular levels of cGMP by activating sGC. Animal studies have shown that iNO therapy improves lung architecture by increasing alveolarization and angiogenesis, and decreasing pulmonary vascular remodeling in the animal models of BPD and/or PH [31, 32]. However, clinical trials have failed to show the effect to a similar extent in prevention and treatment of BPD, and iNO is also exceedingly expensive and cannot be used in non-ventilated or outpatient environments. Therefore, the current consensus is to limit the use of iNO to late preterm and term infants with PH [33] and not to use it routinely as an early rescue treatment in premature babies. On the other hand, sildenafil increases intracellular cGMP levels by inhibiting PDE5. The current widespread off-label use of sildenafil is based on various neonatal animal studies and retrospective clinical studies showing improvement in alveolar development, vascular density, PH and right ventricular function [34–37]. However, data on long term outcomes of chronic sildenafil therapy in infants with BPD-associated PH are lacking and recently, the FDA has issued a warning against sildenafil use in children after a study showed higher mortality in children taking higher doses of sildenafil [13].

Dysregulation of the NO-sGC-cGMP pathway in PH is characterized by diminished bioavailability of NO, reduced sensitivity of sGC to NO, impairment of cGMP production and increased PDE5 activity leading to decreased cGMP levels [38–40]. Therefore, many infants do not respond to iNO or sildenafil treatments [41–43]. Soluble GC stimulators and activators were developed to target those non responders who have low NO bioavailability or have developed tolerance to NO. Riociguat, is a sGC stimulator, which acts synergistically with NO as well as in a NO-independent fashion to stimulate sGC directly causing increased endogenous cGMP production [14]. This alternative mechanism of action suggests riociguat could be a promising alternative or adjuvant therapeutic agent, particularly for those who don’t respond to NO or sildenafil and who need continuation of therapy even after discharge.

This study provids direct evidence that riociguat is an effective agent that prevents hyperoxia-induced BPD. One of the characteristic pathological hallmarks of the “new BPD” in preterm infants is a developmental arrest of immature lungs with simplification of alveoli [1]. Our model showed simplification of alveoli in hyperoxia-exposed/placebo treated animals, but treatment with riociguat largely prevented the poor alveolar growth and development induced by hyperoxia. Further we demonstrated that this improved alveolarization is accompanied by increased cGMP in hyperoxia-exposed lungs, suggesting that the beneficial effect of riociguat on alveolar development is at least in part due to augmentation of cGMP production. In support of this hypothesis, several studies have described the pivotal role of cGMP modulators in alveolarization in normal and injured neonatal lungs [44, 45]. In a 100% oxygen-induced neonatal lung injury model, treatment with Apelin, a potent vasodilator, increased lung cGMP and improved alveolarization [44]. In mouse models, knockout of the sGC-α1 gene leads to decreased cGMP, along with decreased normal alveolarization and worsened alveolar development when exposed to milder hyperoxia (70%) [45].

Impaired angiogenesis is another key feature of BPD in preterm infants. We and others have previously shown that hyperoxia exposure in neonatal rats not only decreases alveolarization, but also reduces vascular development [22, 23, 44]. There is clear evidence that the NO-sGC-cGMP pathway plays an important role in endothelial function and angiogenesis [46, 47]. It acts as one of the second messengers pathways that mediates vascular endothelial growth factor (VEGF) induced proliferation and *in vitro* angiogenesis in human umbilical vein endothelial cells [46]. NO also enhances angiogenesis by increasing VEGF production [47]. In hyperoxic models, sildenafil promotes angiogenesis *in vitro* in human pulmonary arterial endothelial cells and *in vivo* in neonatal rat lungs [48]. Apline also improves vascular growth in hyperoxia-exposed newborn rats [44]. Similarly, we found that stimulation of the NO-sGC-cGMP pathway by administration of riociguat significantly prevented the decrease in rat lung vascular density caused by hyperoxia exposure.

In preterm infants, severe BPD is often complicated by PH which remains a significant cause of mortality and morbidity [7]. The vascular pathology of PH in BPD consists of both decreased vascularization and increased pulmonary vascular remodeling [4–6]. The pulmonary vascular remodeling is characterized by increased muscularization and wall thickness in the peripheral vessels due to an increase in vascular wall smooth muscle cells and their extracellular matrix products. These vascular changes lead to increased pulmonary vascular resistance and RVH. However, we found that riociguat prevented PH in our hyperoxia-exposed neonatal rat model and did so by reducing RVSP and RVH and that these functional changes were accompanied by increased vascular development and decreased peripheral pulmonary vascular remodeling. The reduced pulmonary vascular remodeling was characterized by reduced muscularization and smooth muscle cell proliferation in peripheral vessels. These findings are consistent with the reported anti-PH activity of riociguat in adult animal models [49, 50], and with reports of the actions of other agents that increase cGMP, such as iNO [35], sildenafil [37], and BAY 41–2272, a novel direct activator of sGC [51] in neonatal models of PH.

CTGF is thought to play an important role in hyperoxia-induced lung changes as its gene and protein expression are greatly increased by hyperoxia exposure and in a hyperoxia-induced BPD with PH model in newborn rats, inhibition of CTGF activity by a CTGF neutralizing antibody drastically decreased PH by improving vascular development and reducing vascular remodeling [18]. Furthermore, in a transgenic mouse model, targeted overexpression of CTGF in alveolar type II epithelial cells induced PH and pulmonary vascular remodeling [22]. Thus, one novel finding of our study is that riociguat significantly reduced the increased CTGF gene and protein expression induced by hyperoxia exposure. Although it is unclear how modulators of cGMP levels regulate CTGF expression in the lung, riociguat has been shown to decrease CTGF expression in progressive cardiac remodeling and failure after myocardial infarction [52]. Similarly, cinaciguat, a sGC activator, has also been shown to decrease CTGF expression and protect against glomerular damage in diabetic rats [53].

Inflammation is implicated to play a key role in the pathogenesis of BPD. Many studies have shown higher concentrations of inflammatory mediators as well as higher number of inflammatory cells in tracheal aspirates from preterm infants with BPD [54–56] as well as animal models of BPD [19, 23]. The role of cGMP in the evolution of inflammatory lung diseases has been well described. Glynos et al showed that administration of BAY 58–2667, a sGC activator, prevented resistive breathing induced lung injury and inflammation [57]. Ahluwalia et al demonstrated that leukocyte rolling and adhesion was inhibited by the prototypical GC stimulator BAY 41–2272, and therefore concluded that sGC played a key anti-inflammatory role by inhibiting leukocyte recruitment [58]. Thus it is not surprising that in this study, we found that riociguat decreased the total number of Mac3 expressing macrophages infiltrating the alveolar airspaces due to hyperoxia exposure. However our observation that riociquat had a preferential suppressive effect on M1 macrophage without reducing the hyperoxia-induced M2 macrophages, may be a novel finding. Previous studies confirm our observation that hyperoxia-induced neonatal lung injury is associated with both M1 and M2 macrophage polarization. In newborn mice, exposure to hyperoxia (100% oxygen) from postnatal day 1 to day 7 exacerbated postnatal inflammation-induced lung Injury and promoted the M1 macrophage phenotype [59]. Also in newborn mice, exposure to >90% oxygen for 5 days increased the numbers of M2-polarized macrophage infiltrating the lung [60]. And at least one recent study confirms our observation that preferential reduction of hyperoxia-induced M1 macrophages improves lung function as it has been demonstrated that mesenchymal stem cell exosomes ameliorate hyperoxia (75% oxygen)–induced BPD by suppressing the pro-inflammatory “M1” state and augmenting an anti-inflammatory “M2-like” stage of alveolar macrophages [61].

Recent studies show that the synthesis and activation of macrophage inflammatory mediators are regulated by inflammasome cascades and there is an increased interest in the role of NLRP inflammasomes in neonatal lung injury, particularly BPD as the NLRP-3 inflammasome has been shown to play a critical role in clinical BPD and hyperoxia-induced BPD in neonatal mice [62]. Similarly, one of our recent studies has shown that inhibition of Rac1 signaling ameliorates hyperoxia-induced BPD and that is associated with down-regulating the NLRP-1 inflammasome and mature IL-1β expression [63]. Our finding that riociguat also inhibited the NLRP-1 inflammasome cascade as demonstrated by decreased release of activated capspase-1 and mature IL-1β in oxygen exposed lungs suggests that the anti-inflammatory activity of riociquat on neonatal lungs exposed to oxygen is due to both down-regulation of M1 macrophage polarization and to inhibition of the NLRP-1 inflammasome/IL-1β cascade.

The NO-sGC-cGMP pathway is also an important regulator of the metabolism and function of osteoblasts and osteoclasts and regulates bone formation, resorption and remodeling [64, 65]. Although, the initial animal studies of riociguat activity in adolescent rats and mice showed various degrees of bone resorption and remodeling in the femur and tibia [17], clinical studies in adults did not show any adverse effect on bones. In addition, we have reported normal bone densities in mice lacking GC1 demonstrating that GC1 does not play an important role in specifying bone density [66]. Regardless, the FDA has raised a concern for a future reference for use of riociguat in pediatric patients, even when conducting clinical trials [67]. However, Homer et al, in their recent study using 7 to 9 weeks old Sprague Dawley rats, demonstrated that while riociguat initially caused adverse bone changes, they found these bone changes reversible, with partial recovery after 2 weeks and no bony changes at all after 5 weeks of recovery [17]. Similarly, in our study, we found that daily IP injection of riociguat for 9 days did not affect trabeculae, bone length and bone volume in secondary spongiosa of femur.

There are potential limitations in directly comparing our study to the neonatal patient situation. First, multiple factors such as infection, inflammation, mechanical ventilation, oxygen toxicity, and prematurity all contribute to the pathogenesis of BPD and PH. In our study, we used an extreme hyperoxia-induced lung injury model in Sprague Dawley newborn rats that primarily represents oxygen toxicity. This high level of oxygen was selected based on its ability to induce a severe BPD phenotype that has PH. We realize that this level of oxygen exposure is not commonly used in preterm infants who are at a risk for developing BPD. Future studies will be conducted to test the efficacy of riociguat in preventing and treating rodent models of BPD that are induced by antenatal inflammation and moderate postnatal hyperoxia, two risk factors representing the “double hits” in preterm infants. Second, the riociguat doses used in this study; while physiological effective, might be unnecessarily high since they were based on published studies in adult animals [68]. Third, during phase 2 and phase 3 trials, riociguat was generally well tolerated in adult human populations with a systemic side effect of only 10% hypotension [69, 70]. However, it was not possible for us to assess systemic hypotension in Sprague Dawley newborn rat pups. It will therefore be important to conduct studies trying different doses and evaluating pharmacokinetic properties as well as systemic side effects for riociguat in larger animal models, such as baboons or lambs with prolonged oxygen exposure and mechanical ventilation. Fourth, it can be argued that sGC stimulators, like riociquat, may be less effective in increasing cGMP in hyperoxic conditions than sGC activators as sGC stimulators reportedly have a limited ability to stimulate oxidized sGC, which theoretically should be increased by hyperoxia [15, 16]. While we did find riociquat increased cGMP and had a protective effect against hyperoxia-induced lung injury, perhaps these beneficial findings could be improved with the use of a sGC activator. Thus, future studies to compare the effectiveness of sGC stimulators vs sGC activators in preventing hyperoxia-induced neonatal lung injury are needed. Additionally, we did not look at the histological effects or the long-term effects of riociguat on bone architecture and therefore, it will be important to study the dose dependent long-term effects of riociguat on bones in future studies.

In conclusion, our study is the first to our knowledge, to describe the therapeutic use of the sGC stimulator, riociguat, in a neonatal rat model of hyperoxia induced BPD and PH, including its effects on bone development. Riociguat enhanced cGMP production and lead to the prevention of hyperoxia induced lung inflammation, alveolar simplification, disrupted vascular growth, vascular remodeling and ultimately ameliorated PH. Furthermore, we did not observe any adverse effects on bone growth. These observations suggest that riociguat may have potential as a novel therapeutic agent to alleviate BPD and PH in neonates.

## Supporting information

S1 Data Data underlying this study. (XLSX)
